# Feasibility and performance of spin-echo EPI MR elastography at 3 Tesla for staging hepatic fibrosis in the presence of hepatic iron overload

**DOI:** 10.1007/s00261-023-04160-0

**Published:** 2024-07-11

**Authors:** David Sgier, Daniel Stocker, Christoph Jüngst, Melanie Renzulli, Hanna Biletska-Hanchorova, Achim Weber, Stephan Kannengiesser, Christoph Gubler, Caecilia S. Reiner

**Affiliations:** 1 Institute of Diagnostic and Interventional Radiology, University Hospital Zurich, Raemistrasse 100, 8091 Zurich, Switzerland; 2 University of Zurich, Zurich, Switzerland; 3 Department of Gastroenterology and Hepatology, University Hospital Zurich, Zurich, Switzerland; 4 Institute of Pathology and Molecular Pathology, University Hospital Zurich, Zurich, Switzerland; 5 MR Application Predevelopment, Siemens Healthcare GmbH, Erlangen, Germany; 6 Department of Gastroenterology and Hepatology, Stadtspital Triemli, Zurich, Switzerland

**Keywords:** Elasticity imaging techniques, Spin-echo EPI MR elastography, Liver cirrhosis, Iron overload

## Abstract

**Purpose:**

To assess the feasibility and performance of MR elastography (MRE) for quantifying liver fibrosis in patients with and without hepatic iron overload.

**Methods:**

This retrospective single-center study analyzed 139 patients who underwent liver MRI at 3 Tesla including MRE (2D spin-echo EPI sequence) and R2* mapping for liver iron content (LIC) estimation. MRE feasibility and diagnostic performance between patients with normal and elevated LIC were compared.

**Results:**

Patients with elevated LIC (21%) had significantly higher MRE failure rates (24.1% vs. 3.6%, *p* < 0.001) compared to patients with normal LIC (79%). For those with only insignificant to mild iron overload (LIC < 5.4 mg/g; 17%), MRE failure rate did not differ significantly from patients without iron overload (8.3% vs. 3.6%, *p* = 0.315). R2* predicted MRE failure with fair accuracy at a threshold of R2* ≥ 269 s^−1^ (LIC of approximately 4.6 mg/g). MRE showed good diagnostic performance for detecting significant (≥ F2) and severe fibrosis (≥ F3) in patients without (AUC 0.835 and 0.900) and with iron overload (AUC 0.818 and 0.889) without significant difference between the cohorts (*p* = 0.884 and *p* = 0.913). For detecting cirrhosis MRE showed an excellent diagnostic performance in both groups (AUC 0.944 and 1.000, *p* = 0.009).

**Conclusion:**

Spin-echo EPI MRE at 3 Tesla is feasible in patients with mild iron overload with good to excellent performance for detecting hepatic fibrosis with a failure rate comparable to patients without iron overload.

**Supplementary Information:**

The online version contains supplementary material available at 10.1007/s00261-023-04160-0.

## Introduction

Traditionally, liver biopsy was considered the reference standard for assessment of liver fibrosis [1]. Considering the known disadvantages of liver biopsy, several non-invasive methods for diagnosing liver fibrosis have been developed [2]. Magnetic resonance elastography (MRE) is currently considered the most accurate non-invasive method for the detection and staging of liver fibrosis. Multiple studies have demonstratated high accuracy for the identification of fibrosis (stage 2 or higher) for MRE [3–8].

MRE can be performed with most MR scanners at 1.5 and 3 Tesla using additional hardware to generate mechanical waves. Increased rigidity of the liver parenchyma can be visualized by modified phase-contrast pulse sequences. The stiffness generated from the wave propagation information by dedicated software (“inversion” of the wave equation) can be depicted on cross-sectional parameter maps ("elastograms") which may allow inference about collagen deposition. MRE mostly uses gradient-echo techniques. However, one of its main limitations is its susceptibility to liver iron overload. Excess iron concentration in the liver degrades signal intensity in MRE sequences, resulting in technical failure and non-diagnostic results [9–12]. Furthermore, this signal degradation is increased at 3 Tesla compared to 1.5 Tesla field strength. Spin-echo echo-planar imaging (SE-EPI) MRE sequences are less susceptible to iron overload. Therefore, we hypothesize that the failure rate below a certain liver iron level, estimated by the multi-gradient-echo relaxation rate R2*, is similar compared to livers without iron overload. To date, there are only few previous studies assessing the correlation between hepatic iron levels and MRE failure [9, 11–14], and no previous study evaluated the diagnostic performance of SE-EPI MRE at 3 Tesla for staging of liver fibrosis in the presence of liver iron overload.

The purpose of this study was to compare the SE-EPI MRE failure rate, image quality and the diagnostic performance between cohorts of patients with normal liver iron levels and with liver iron overload. Additionally, our objective was to identify an R2* threshold value at which our SE-EPI MRE protocol demonstrates an acceptable rate of success.

## Materials and methods

### Patients

This single-center retrospective study was approved by the institutional review board and local ethics committee. A general informed consent for retrospective data evaluation was signed by all study participants.

Our clinical database was queried to identify patients who underwent liver MRI including MRE at our institution between August 2015 and March 2021. Inclusion criteria were as follows: (1) men or women 18 years of age or older, (2) participants with suspected or known chronic liver disease undergoing liver MRI including MRE, (3) MRI-based liver iron assessment, and (4) signed written informed consent form. The exclusion criteria were as follows: (1) neither confirmation of fibrosis stage nor hepatic iron overload, (2) incomplete scan protocols, (3) active driver error of MRE.

**Fig. 1:**
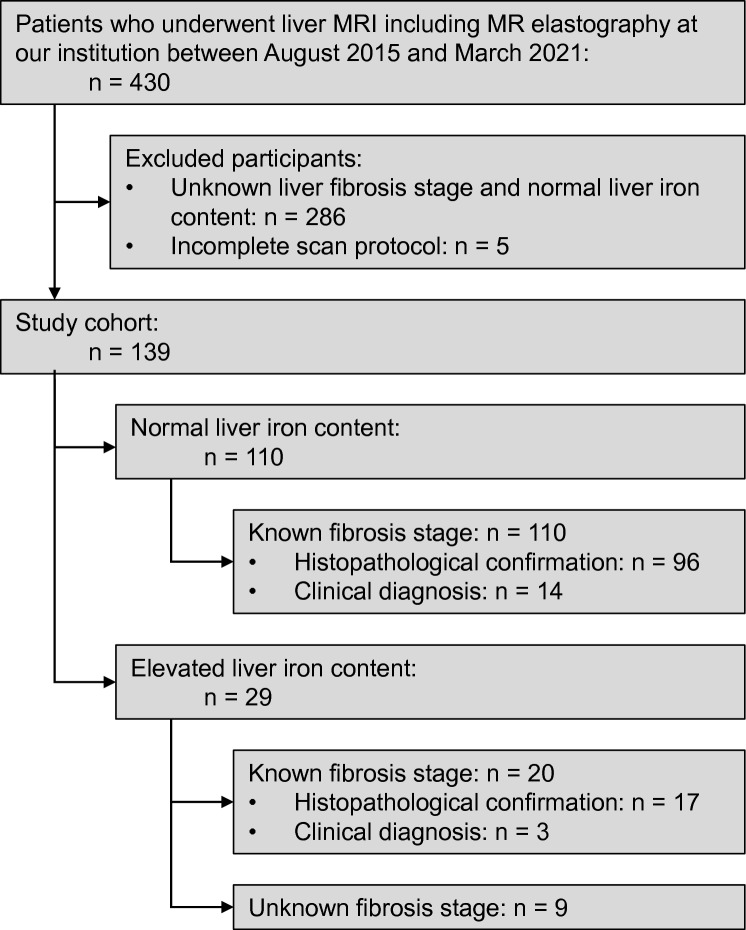
Flowchart of the patient selection process. MRE, MR elastography

A total of 430 data sets was extracted from our database. 286 patients were excluded due to neither confirmed liver fibrosis stage nor hepatic iron overload, and 5 patients due to incomplete scan protocols. The final study cohort consisted of 139 patients (Fig. 1). 120 patients were part of a prospective study on multiparametric MRI in diffuse liver disease, whereof 68 patients have been reported previously [15]. The liver fibrosis stage was known in 130 of 139 patients (94%) with histopathological confirmation in 113 patients (81%) and clinical diagnosis of cirrhosis based on clinical and imaging signs of cirrhosis and concomitant portal hypertension in 17 patients (12%). In all patients without hepatic iron overload and in 20 of 29 patients with hepatic iron overload the hepatic fibrosis stage was confirmed. All liver biopsies were clinically indicated and the methodology for the histological analysis of the liver specimen was described elsewhere [16].

### Liver MRI data acquisition

All MR liver examinations were performed on the same 3 Tesla clinical MRI system (MAGNETOM Skyra; Siemens Healthineers) with an 18-channel body coil in combination with a built-in 32-channel spine coil, or a 60-channel body coil. The MRI protocol included the following imaging sequences for anatomical correlation: T2-weighted HASTE coronal, T2-weighted fat-saturated HASTE axial, non-contrast dual-gradient-echo T1-weighted axial with Dixon reconstruction. For liver iron assessment, a 3D multi-gradient-echo sequence, covering the entire liver, in axial plane was acquired as described previously [15] and R2* maps for liver iron content (LIC) estimation (Multi-Echo Dixon VIBE from LiverLab product option) were derived. MRE data acquisition was performed using a 2D spin-echo sequence with a fast echo-planar imaging (2D SE-EPI) readout on four slices through the midsection of the liver with the following imaging parameters: TR, 1400 ms; TE, 47 ms; FOV 384 mm; matrix size, 256 × 256; slice thickness, 6 mm, 4 slices; bandwidth, 2380 Hz/pixel; number of averages, 1; motion encoding gradient duration 16.67 ms; EPI factor 100. The first 18 patients were scanned with TR, 1500 ms and TE, 50 ms and 5 slices. The passive driver of the acoustic wave generator (Resoundant Inc.) was placed on the right upper quadrant of the abdominal wall at the level of the xiphisternum, centered on the mid-clavicular line propagating 60-Hz pneumatic vibrations to the liver. MRE was acquired in expiration breath hold (duration 15 s). The scanner automatically generated magnitude, phase difference, and wave images as well as an elastogram including a 95% confidence map of the MRE.

### Qualitative analysis of MRE

One board-certified abdominal radiologist (D.St., 7 years of experience), blinded to the clinical patient data and estimated LIC, qualitatively evaluated the MRE data sets. An MRE was defined as successful in case of visible varying waves in the liver on the phase images that produced measurable data within the bounds of the elastogram confidence maps. Failed MRE was defined as no visible waves, disordered, or fragmented waves in the liver on the wave images with no measurable area of liver within the bounds of the elastogram confidence maps.

The quality of wave propagation was rated as (0) no visible waves (technical failure), (1) barely visible waves, (2) clearly visible waves with two or fewer wavelength penetrating the liver, and (3) clearly visible waves with more than two wavelengths penetrating the liver.

Furthermore, patient related factors potentially decreasing MRE quality were assessed (ascites, morphological cirrhosis, lung tissue between liver and driver, active driver error, breathing artifacts, see Supplementary Table 1).

### Quantitative analysis of MRE and R2*

Quantitative image analysis was performed in *syngo.*via (version VB20, Siemens Healthineers).

MRE quality was quantitatively assessed by measuring the percentage of measurable liver area on the stiffness maps with a confidence map value equal or greater than 95% (C95). Therefore, for each data set, a free-hand region of interest (ROI) was placed on each of the four magnitude images and the corresponding C95-stiffness maps. The objective was to encompass the maximum amount of liver parenchyma while avoiding large vessels and central bile ducts and keep a 1 cm margin from the liver edges.

The percentage of measurable liver area was determined by calculating the ratio of the size of the ROI on the C95-stiffness maps to the total area of the liver on the corresponding magnitude image for each of the four slices. This was then calculated as the mean over the four slices for each data set. In addition, the following parameters were measured on the magnitude images: Maximum distance between the skin and liver surface at the level of the passive driver (in cm), the maximal abdominal diameter from right to left and from anterior to posterior at the level of the passive driver (in cm).

Liver stiffness (LS) was measured on the stiffness maps (in kilopascals, kPa) by placing one polygon ROI within the borders of the liver on each of the four slices in conjunction with the magnitude and wave images. ROIs were drawn by one trained observer (D.Sg.) to be as large as possible while remaining within the margins of the confidence map, avoiding major blood vessels, regions close to the driver and remaining approximately 1 cm inside the boundary of the liver. To determine the overall LS, we computed the mean LS value across all slices obtained from each ROI [17]. Additionally, we calculated the weighted LS by taking the mean LS values from each slice, weighted according to the size of the corresponding ROI [18].

R2* values (in s^−1^) were obtained from the R2* maps by manually drawing a single, free-hand ROI in one slice, covering as much of the liver as possible while excluding large vessels and artifacts. Hepatic iron overload was defined as an R2* value equal or greater than 115 s^−1^. We subdivided the patient cohort with iron overload into two groups, based on the severity of their condition as proposed by Henninger et al. [19]: Patients with insignificant to mild iron overload (R2* values ranging from 115 to less than 320 s^−1^), and patients with moderate to severe iron overload (R2* values ≥ 320 s^−1^).The estimated LIC in mg iron per gram of liver dry weight was calculated using the following formula: LIC = 0.017 mg Fe/g *X* R2* *X* s [19].

### Statistical analysis

Patient characteristics and MRI values were summarized with descriptive statistics. Appropriate statistical tests were used for the comparison of variables. Generalized linear regression analyses were used to identify independent predictors of MRE image quality and measurable liver area. Spearman rank correlations were used to test the correlation between non-metrical variables. Correlation coefficients were classified as follows: 0–0.19 no or negligible relationship, 0.20–0.29 weak, 0.30–0.39 moderate, 0.40–0.69 strong, and ≥ 0.7 very strong [20, 21]. Receiver operating characteristic (ROC) analysis and the Youden index were utilized to assess the diagnostic performance of MRE for diagnosing liver fibrosis stage and identify an R2* threshold to predict MRE failure. ROC analyses were performed only for patients with confirmed stage of hepatic fibrosis (*n* = 131). The ROC analysis was run for the entire sample as well as separately for the patients with normal vs. those with elevated LIC and compared using bivariate statistical analysis (Χ^2^ test) [22]. AUC values < 0.6 indicated no accuracy, 0.61–0.7 poor accuracy, 0.71–0.8 fair accuracy, 0.81–0.9 good accuracy, and 0.91–1.00 excellent accuracy [8, 23]. A *p*-value of less than 0.05 was considered statistically significant. All statistical tests were two-sided. Statistical analyses were performed using SPSS (version 28, IBM Corporation).

## Results

### Patient and liver MRI characteristics

In total, 139 subjects were included in this study: 110 (79.1%) with normal estimated LIC (median R2* 52.4 s^−1^, range 24.7–111.3 s^−1^) and 29 (20.9%) with iron overload (median R2* 170.9 s^−1^, range 115.7–1058.4 s^−1^; median 2.9 mg Fe/g dry weight). Of the 29 patients with iron overload 24 had insignificant to mild iron overload (median R2* 155.5 s^−1^, range 115.7–271.4 s^−1^; median 2.6 mg Fe/g dry weight) and 5 had moderate to severe iron overload (median R2* 529.4 s^−1^, range 385.0–1058.4 s^−1^; median 9.0 mg Fe/g dry weight). A summary of patient characteristics is presented in Table 1.

**Table 1:** Patient characteristics for the overall cohort and for patients with and without iron overload

	All patients (*n* = 139)	Normal iron concentration (*n* = 110)	Iron overload (*n* = 29)	*p* value
Age (years)	49.2 ± 13.8	48.5 ± 13.3	51.9 ± 15.5	0.250
*Sex*				0.002
Female	43 (31%)	41 (37%)	2 (7%)	
Male	96 (69%)	69 (63%)	27 (93%)	
Body mass index (kg/m^2^)	27.7 ± 5.1	28.2 ± 5.4	25.8 ± 3.6	0.025
Height (cm)	173 ± 9	172 ± 9	176 ± 8	0.029
Body weight (kg)	83.4 ± 17.9	84.2 ± 19.0	80.5 ± 13.1	0.332
*Underlying liver disease*				< 0.001
ALD	26 (19%)	19 (17%)	7 (24%)	
NAFLD	40 (29%)	34 (31%)	6 (21%)	
Hepatitis B	20 (14%)	20 (18%)	0 (0%)	
Hepatitis C	10 (7%)	9 (8%)	1 (3%)	
Hemochromatosis	6 (4%)	0 (0%)	6 (21%)	
Autoimmune hepatitis	6 (4%)	6 (5%)	0 (0%)	
PSC	3 (2%)	3 (3%)	0 (0%)	
PBC	2 (1%)	1 (1%)	1 (3%)	
Wilson’s disease	1 (1%)	1 (1%)	0 (0%)	
Other	9 (6%)	7 (6%)	2 (7%)	
Hepatopathy of unknown etiology	14 (10%)	9 (8%)	5 (17%)	
Data unavailable	2 (1%)	1 (1%)	1 (3%)	
*Stage of liver fibrosis*^a^				0.707
0	14 (11%)	11 (10%)	3 (15%)	
1	26 (20%)	23 (21%)	3 (15%)	
2	32 (25%)	29 (26%)	3 (15%)	
3	27 (21%)	22 (20%)	5 (25%)	
4	31 (24%)	25 (23%)	6 (30%)	

The sample mean ± standard deviation is given for continuous variables. For the comparison of variables between the subsets of patients with and without iron overload, X^2^, independent samples t-test or Mann–Whitney test were used for the calculation of *p* values

*ALD* alcohol-related liver disease, *NAFLD* nonalcoholic fatty liver disease, *PSC* primary sclerosing cholangitis, *PBC* primary biliary cholangitis

^a^Only patients with known liver fibrosis stage

### Comparison of MRE feasibility between patients with normal vs patients with elevated LIC

**Fig. 2:**
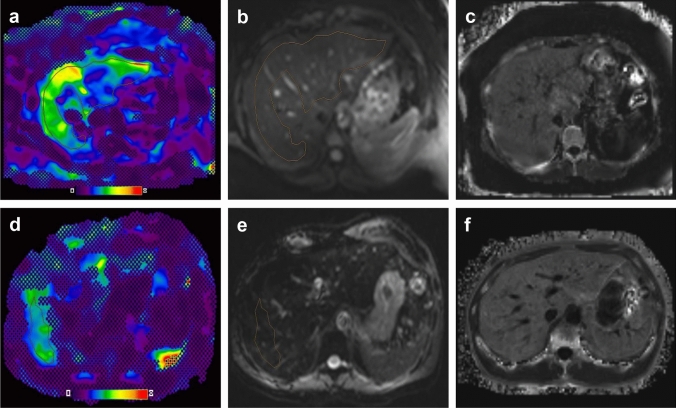
**a**–**c** A 26-year old female with NAFLD, BMI = 40.4 kg/m2, fibrosis stage F3. **a** MR elastography shows a mean stiffness of 3.40 kPa as outlined on the C95-stiffness map (**a**) and magnitude image **b** correctly identifying severe fibrosis. The mean measurable liver area over the four slices of the C95-stiffness map was 27%. **c** The measured R2* value was 66 s^−1^. **d**–**f** A 60-year old male with hepatitis C, BMI = 21.3 kg/m^2^, fibrosis stage F3. **d** MR elastography shows a mean stiffness of 3.09 kPa as outlined on the C95-stiffness map (**d**) and magnitude image (**e**) correctly identifying severe fibrosis. The mean measurable liver area over the four slices of the C95-stiffness map was 5%. **c** The measured R2* value was 135 s^−1^

Out of 139 MRE examinations, 128 (92.1%) were successful and 11 (7.9%) unsuccessful (Table 2). The MRE failure rate was significantly higher in patients with elevated LIC compared to patients with normal LIC (24.1% vs. 3.6%, *p* < 0.001). Both, the quality of wave propagation and the measurable liver area on MRE confidence maps were significantly reduced in patients with elevated LIC (Table 2, Fig. 2).

**Table 2:** Magnetic resonance elastography characteristics for patients with normal liver iron, insignificant to mild iron overload and moderate to severe iron overload

	Normal iron concentration (*n* = 110)	Insignificant to mild iron overload (*n* = 24)		Moderate to severe iron overload (*n* = 5)
MRE failure rate	3.6% (4)	8.3% (2)		100.0% (5)
	*p* = 0.315			
			*p* < 0.001	
*Quality of wave propagation (% of MRE)*				
0	2.7% (3)	4.2% (1)		40.0% (2)
1	1.8% (2)	4.2% (1)		60.0% (3)
2	8.2% (9)	16.7% (4)		0.0% (0)
3	87.3% (96)	75.0% (18)		0.0% (0)
	*p* = 0.133			
			*p* < 0.001	
Mean percentage of measurable liver area^a^	34.5% ± 10.3%	22.9% ± 14.0%		-
	*p* < 0.001			
			-	

Normal iron concentration: R2* < 115 s^−1^. Insignificant to mild iron overload: 115 s^−1^ ≤ R2* < 320 s^−1^. Moderate to severe iron overload: R2* ≥ 320 s^−1^. For the calculation of *p* values, the X^2^ (MRE failure rates), Mann–Whitney test (quality of wave propagation), and the independent samples t-test (measurable liver area) were used. Quality of wave propagation: (0) no visible waves (technical failure), (1) barely visible wave propagation, (2) clearly visible waves with two or fewer wavelength penetrating the liver, and (3) clearly visible waves with more than two wavelengths penetrating the liver

^a^Successful elastograms only

A predictive model for estimating measurable liver area and quality of wave propagation using generalized linear regression with factors potentially influencing MRE quality showed that R2*, underlying liver disease, lung tissue between liver and driver, body mass index, and abdominal diameter were independent and significant predictors of measurable liver area. R2*, underlying liver disease, and active driver error were independent predictors of quality of wave propagation (Table 3). The strongest patient related predictors for measurable liver area and quality of wave propagation were R2* and underlying liver disease.

**Table 3:** Univariate generalized linear regression analyses for predictors of measurable liver area and quality of wave propagation

Factor	Wald *X*^2^	*p* value
*Measurable liver area*		
R2* (s^−1^)	19.57	< 0.01
Age (years)	2.59	0.11
Sex	0.03	0.86
Body mass index (kg/m^2^)	9.53	< 0.01
Underlying liver disease	29.52	< 0.01
Stage of liver fibrosis	8.00	0.09
Amount of ascites	0.33	0.57
Liver morphology	0.76	0.68
Lung tissue	5.08	0.02
Breathing artifacts	0.02	0.88
Max. distance between skin and liver beneath the driver	0.23	0.63
Abdominal diameter (left to right, cm)	4.40	0.04
Abdominal diameter (anterior to posterior, cm)	6.79	0.01
*Quality of wave propagation*		
R2* (s^−1^)	18.99	< 0.01
Age (years)	0.29	0.59
Sex	0.87	0.35
Body mass index (kg/m^2^)	0.22	0.64
Underlying liver disease	20.59	0.01
Stage of liver fibrosis	5.46	0.24
Amount of ascites	0.02	0.88
Liver morphology	0.58	0.75
Lung tissue	1.87	0.17
Driver error	65.84	< 0.01
Breathing artifacts	0.07	0.79
Max. distance between skin and liver beneath the driver	0.00	0.95
Abdominal diameter (left to right, cm)	0.08	0.78
Abdominal diameter (anterior to posterior, cm)	1.17	0.28

Patients with normal LIC and insignificant to mild iron overload showed comparable MRE failure rates (3.6% vs. 8.3%, *p* = 0.315) and quality of wave propagation (Table 2). The measurable liver area on the elastogram confidence maps was significantly smaller in patients with insignificant to mild iron overload compared to patients with normal LIC (34.5% ± 10.3% vs. 22.9% ± 14.0%, *p* < 0.001). All MREs in patients with moderate to severe iron overload failed (*n* = 5).

The measurable liver area in patients incorrectly classified regarding their degree of fibrosis was similar to the measurable liver area in patients correctly classified (34% ± 8.5% vs. 34.5% ± 10.9% in patients without iron overload; 27.0% ± 12.3% vs. 23.9% ± 16.4% in patients with iron overload). There was no relevant correlation between the accurate classification of liver fibrosis by MRE and the measurable liver area, for the entire patient cohort (Spearman’s *r* = 0.001, *p* = 0.995), as well as when looking at cases with normal iron levels (Spearman’s *r* = − 0.003, *p* = 0.973) and cases with iron overload separately (Spearman’s *r* = − 0.088, *p* = 0.746).

### Prediction of MRE failure by R2* and identification of R2* cut-off value

**Fig. 3:**
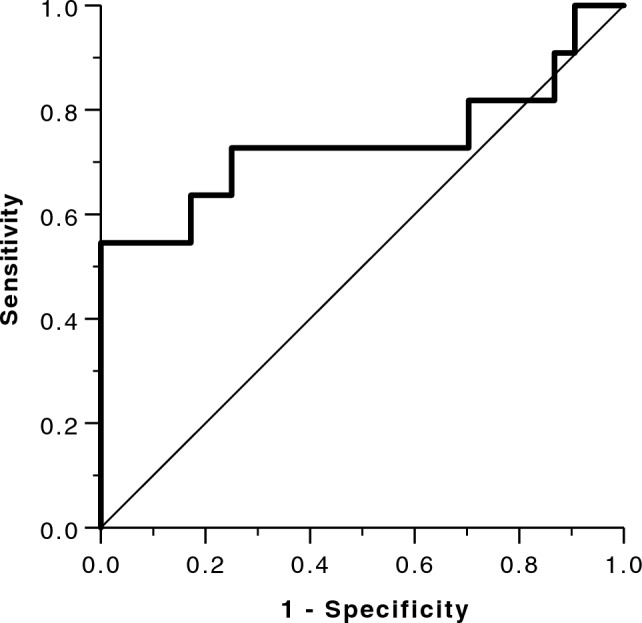
Receiver operator characteristics curve for the prediction of SE-EPI MRE failure by R2* values

R2* predicted MRE failure with fair accuracy (AUC 0.737, 0.525–0.948 95%CI, *p* = 0.009) using a threshold of R2* ≥ 269 s^−1^ (Fig. 3). In all patients (*n* = 6) with R2* ≥ 269 s^−1^ (≥ 4.6 mg Fe/g dry weight) MRE failed. Applying this threshold would result in an MRE failure rate of 4.3% (1/23 patients) for patients with mild iron overload.

### Diagnostic performance for staging of liver fibrosis by MRE

**Fig. 4:**
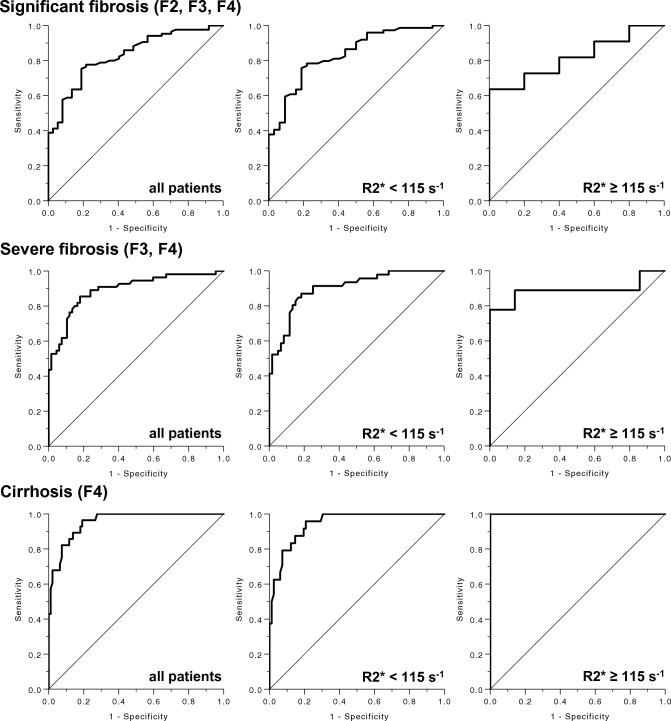
Receiver operator characteristics curves illustrating the ability of SE-EPI MRE to predict significant fibrosis, severe fibrosis, and cirrhosis in the total cohort (first column), patients without liver iron overload (second column) and patients with liver iron overload (third column)

For detecting significant (≥ F2) and severe fibrosis (≥ F3) MRE showed good diagnostic performance in patients without and patients with iron overload without significant difference between the cohorts (*p* = 0.884 and *p* = 0.913). For detecting cirrhosis MRE showed an excellent diagnostic performance in both groups (*p* = 0.009). Details regarding the diagnostic performance for staging of liver fibrosis by MRE are presented in Table 4 and Fig. 4.

**Table 4:** Receiver operating characteristic curve analysis for the prediction of liver fibrosis by MR elastography

Cohort	Liver stiffness threshold (kPa)	Area under the curve^a^	Sensitivity	Specificity	*p* value	*p* value
*Significant fibrosis (F2, F3, F4)*						
All patients	≥ 2.73	0.831 (0.758—0.904)	74.1%	81.1%	< 0.001	
R2* < 115 s^−1^	≥ 2.73	0.835 (0.756—0.914)	75.7%	81.2%	< 0.001	0.884
R2* ≥ 115 s^−1 b^	≥ 3.01	0.818 (0.610—1.000)	63.6%	100.0%	0.047	
*Severe fibrosis (F3, F4)*						
All patients	≥ 3.03	0.893 (0.834—0.952)	85.5%	82.1%	< 0.001	
R2* < 115 s^−1^	≥ 3.11	0.900 (0.841—0.958)	87.0%	81.7%	< 0.001	0.913
R2* ≥ 115 s^−1 b^	≥ 3.01	0.889 (0.705—1.000)	77.8%	100.0%	0.001	
*Cirrhosis (F4)*						
All patients	≥ 3.54	0.954 (0.920—0.988)	96.4%	80.9%	< 0.001	
R2* < 115 s^−1^	≥ 3.54	0.944 (0.902—0.986)	95.8%	79.3%	< 0.001	0.009
R2* ≥ 115 s^−1 b^	≥ 4.86	1.000 (1.000—1.000)	100.0%	100.0%	0.004	

Cohort: includes only patients with confirmed hepatic fibrosis stage; all patients, *n* = 131; R2* < 115 s^−1^, *n* = 111; R2* ≥ 115 s^−1^, *n* = 20

^a^Data in brackets are 95% CIs

^b^Includes only patients with insignificant to mild iron overload

## Discussion

In our study, the overall MRE failure rate at 3 Tesla was 7.9% with a significantly higher failure rate in patients with elevated liver iron (24.1%) compared to patients with normal liver iron (3.6%). We found comparable MRE failure rates between patients with normal estimated LIC and patients with insignificant to mild iron overload. However, MRE failed in all patients with moderate to severe iron overload. Furthermore, we were able to show that R2* predicted SE-EPI MRE failure at 3 Tesla with fair accuracy using a threshold of R2* ≥ 269 s^−1^ (corresponding to a LIC of approximately ≥ 4.6 mg/g dry weight).

In a previous analysis of 1377 scans using gradient-echo (gre-) MRE on both 1.5 and 3 Tesla scanners, Yin et al. reported a failure rate of 5.6%, with iron overload accounting for over 70% of cases where MRE was not diagnostic [10]. In another study, Wagner and colleagues reported a failure rate of 15.3% at 3 Tesla and 3.5% at 1.5 Tesla for gre-MRE [9]. The authors concluded that the technical failure rate for gre-MRE was low at 1.5 Tesla but substantially higher at 3 Tesla. They identified iron deposition as independent factors associated with MRE failure. For SE-EPI based MRE failure rates of 1.9%, 2.8% and 4% at 3 Tesla in patients with chronic liver disease have been reported [24–26]. These failure rates are considerably lower than in our cohort, which can in part be explained with the lower rate of patients with elevated LIC (2.8% and 6.5%) compared to our patient cohort (21%) [24, 25].

The feasibility of SE-EPI-MRE in patients with iron overload has been studied by Serai and colleagues at 1.5 T [12]. While they were able to show the feasibility of measuring liver stiffness with MRE in patients with elevated LIC, they reported a high failure rate of 23% [12]. This failure rate is similar to our failure rate of 24% at 3 Tesla in patients with a lower mean iron overload in our cohort (estimated mean liver iron content 9.3 mg Fe/g [12] vs. 4.3 mg Fe/g liver dry weight). A similar failure rate of 21% for MRE in patients with iron overload was found by a study using a SE-EPI-MRE sequence on a 3 Tesla scanner from a different vendor [13]. In another study, which investigated the effect of R2* on different MRE sequence types at 3 Tesla, the failure rate of a SE-EPI-MRE sequence similar to our sequence was 6.3% in patients with mild iron overload, which compares to the failure rate of 8.3% in our cohort with mild iron overload [14].

Ghoz et al. established a T2* cut-off of 20 ms (corresponding to a liver iron concentration of 1.5 mg Fe/g dry weight) for gre-MRE at 1.5 Tesla, which effectively predicted non-diagnostic MRE results [11]. We determined an R2* threshold value of 269 s^−1^ (corresponding to an LIC of approximately 4.6 mg Fe/g dry weight) below which our SE-EPI-MRE protocol at 3 Tesla yielded results for individuals with mild iron overload that were comparable to those without iron overload. Plaikner et al. [27] identified a maximum R2* of 375 s^−1^ for partially usable SE-EPI-MRE stiffness maps and a maximum R2* of 563 s^−1^ for hiSE-EPI MRE at 1.5 Tesla in a cohort of patients with and without iron overload. The higher maximum R2* value compared to our maximum R2* value can be partly explained by differences in the SE-EPI MRE sequence type: their hiSE-EPI MRE variant had shorter echo time through fractional encoding, which is less susceptible to iron, and in addition thicker slices and 3 averages, which will improve signal to noise ratio. The median LIC in patients with iron overload (approximately 3.6 mg Fe/g) in this study was similar to our patients with iron overload.

In our study, the quality of wave propagation and the measurable liver area on the MRE confidence maps were significantly reduced in patients with elevated vs. normal liver iron levels. These findings are in line with the study by Felker et al. [26], who reported a significant inverse correlation of liver R2* and measureable liver area for SE-EPI MRE and gre-based MRE and a significantly higher liver R2* in patients with failed MRE. Also, a recent smaller study, comparing SE-EPI MRE and gre-MRE at 3 Tesla reported a smaller measureable liver area on LS maps in patients with iron overload than in patients without iron overload [28].

In our entire study population, MRE effectively differentiated between various stages of fibrosis with a high degree of diagnostic accuracy, consistent with results reported in the literature [8, 10, 29]. Our results for staging liver fibrosis were similar to previous results with an AUC of 0.83 (0.82–0.87 [8, 10, 29]) for ≥ F2; 0.89 (0.87–0.91 [8, 10, 29]) for ≥ F3; and 0.95 (0.87–0.89 [8, 10, 29]) for F4. In patients with mild iron overload, MRE was able to identify significant (≥ F2) and severe (≥ F3) fibrosis with good accuracy (AUC 0.82 and 0.89 respectively), albeit with a wide 95% confidence interval. While this is on the lower end of the spectrum when compared to the diagnostic performance of MRE in previous studies [8, 10, 29], it still demonstrates the potential of MRE as a reliable diagnostic tool. This finding may be explained by the significantly smaller measurable liver area on MRE LS maps in patients with liver iron overload. Another explanation might be the small sample size of this patient group.

In addition, our liver stiffness thresholds for detection of fibrosis stages ≥ F2 (2.73 kPa), ≥ F3 (3.03 kPa), and F4 (3.54 kPa) lie well within a range of values published in the relevant literature (≥ F2: 2.23–3.80 kPa [7, 8, 29, 30]; ≥ F3: 2.63–4.30 kPa [7, 8, 29, 30]; F4: 2.75–4.70 kPa [7, 8, 29, 30]).

Our study has some limitations. First, the study population was rather heterogeneous with different underlying liver disease. However, a heterogenous population reflects daily clinical practice. Therefore, we believe that our results can be applied in various clinical settings where MRE is indicated. Second, the number of patients with iron overload was small limiting the statistical power of the analysis of diagnostic performance of MRE. Third, we did not use a previously proposed modified SE-EPI MRE sequence with shorter TE based on "fractional encoding", which can further limit the iron-induced signal loss in patients with higher iron overload [27]. This sequence variant is currently not clinically available on the studied scanner. Furthermore, MRE is not limited to a specific type of pulse sequence and can even be performed with a conventional spin-echo sequence reducing the sensitivity to R2* decay, although requiring longer acquisition times with potential impact on image quality in the upper abdomen. Fourth, the study was conducted at a single institution on a single scanner with a specific protocol. Thus, our results may not be directly transferable to other scanner platforms.

In conclusion, SE-EPI MRE at 3 Tesla is feasible in patients with mild iron overload and shows good to excellent performance for detecting severe hepatic fibrosis and cirrhosis with a failure rate comparable to a cohort of patients without iron overload. Further studies in a larger patient population on different scanner systems are needed to confirm these findings.

## Supplementary Information

Below is the link to the electronic supplementary material.

Supplementary file1 (DOCX 14 kb)

## Abbreviations

ALD: Alcohol-related liver disease
AUC: Area under the curve
EPI: Echo-planar imaging
LIC: Liver iron content
LS: Liver stiffness
MRE: MR elastography
NAFLD: Nonalcoholic fatty liver disease
ROC: Receiver operating characteristics
SE: Spin-echo

## Declarations

### Study subjects or cohorts overlap

Some study subjects or cohorts have been previously reported in S. von Ulmenstein, S. Bogdanovic, H. Honcharova-Biletska, S. Blümel, A.R. Deibel, D. Segna, C. Jüngst, A. Weber, T. Kuntzen, C. Gubler, C.S. Reiner, Assessment of hepatic fibrosis and inflammation with look-locker T1 mapping and magnetic resonance elastography with histopathology as reference standard, Abdom Radiol (NY) (2022).
