# Supplementary material for: Feasibility and performance of spin-echo EPI MR elastography at 3 Tesla for staging hepatic fibrosis in the presence of hepatic iron overload
Source: Abdom Radiol (NY). 2024 Jul 11;49(11):3871–82. doi: 10.1007/s00261-023-04160-0 (PMC11519119; doi:10.1007/s00261-023-04160-0)
Supplement: Supplementary file 1 — Supplementary file1 (DOCX 14 kb) [file 261_2023_4160_MOESM1_ESM.docx]

**Supplementary Table 1**: Assessment of additional factors potentially decreasing MRE quality.

| Categorization of variables | |
| --- | --- |
| Amount of ascites  0  1  2  3 | no ascites  minimal perihepatic or perisplenic fluid  moderate intraperitoneal fluid without abdominal wall distension  massive intraperitoneal fluid with abdominal wall distension |
| Liver morphology  0  1  2 | normal, non-cirrhotic morphology  indeterminate morphology, liver without clear nodular contour, but with some morphologic changes (lobar hypertrophy or atrophy)  cirrhotic liver (including enlarged caudate lobe, nodular contour, with or without signs of portal hypertension) |
| Lung tissue  Yes  No | Lung tissue between liver parenchyma and driver  No lung tissue between liver parenchyma and driver |
| Driver error  Yes  No | Driver inactive during scan  Driver active during scan |
| Breathing artefacts  Yes  No | Breathing artefacts  No breathing artefacts |

**Supplementary Table 2**: MR elastography characteristics for the overall cohort as well as for the subsets of patients with and without iron overload.

|  | All patients  (n = 139) | Normal iron concentration  (n = 110) | Iron overload  (n = 29) | P value |
| --- | --- | --- | --- | --- |
| Weighted liver stiffness (kPa) | 3.54 ± 1.81 | 3.56 ± 1.77 | 3.47 ± 2.00 | 0.850 |
| Maximal distance between skin and liver beneath the driver (cm) | 3.2 ± 1.0 | 3.2 ± 1.1 | 3.0 ± 0.9 | 0.277 |
| Abdominal diameter (left to right, cm) | 34.4 ± 3.6 | 34.3 ± 3.7 | 34.4 ± 2.8 | 0.884 |
| Abdominal diameter (anterior to posterior, cm) | 24.8 ± 3.7 | 25.0 ± 3.9 | 24.3 ± 3.0 | 0.301 |
| Amount of ascites  0/1 (no or minimal)  2/3 (moderate or massive) | 136 (98%)  3 (2%) | 108 (98%)  2 (2%) | 28 (97%)  1 (3%) | 0.761 |
| Liver morphology  0/1 (normal or indeterminate)  2 (cirrhotic) | 107 (77%)  32 (23%) | 83 (75%)  27 (25%) | 24 (83%)  5 (17%) | 0.691 |
| Number of elastograms with lung tissue between liver and driver | 3 (2%) | 3 (3%) | 0 (0%) | 0.369 |
| Number of elastograms with active driver error | 2 (1%) | 2 (2%) | 0 (0%) | 0.465 |
| Number of elastograms with breathing artefacts | 5 (4%) | 5 (5%) | 0 (0%) | 0.505 |
| Normal iron concentration: R2* < 115 s^-1^  Iron overload: R2* ≥ 115 s^-1^  The sample mean ± standard deviation is given for continuous variables. For the comparison of variables between patients without and with iron overload, X^2^ test (lung tissue between liver and driver, active driver error, breathing artefacts), Mann-Whitney (amount of ascites, liver morphology) or independent samples t-test (weighted liver stiffness, distance measurements) were used for the calculation of p values. | | | | |
